# Leptin enhances social motivation and reverses chronic unpredictable stress-induced social anhedonia during adolescence

**DOI:** 10.1038/s41380-022-01778-2

**Published:** 2022-09-22

**Authors:** Yun Lei, Dan Wang, Yu Bai, Jayvon Nougaisse, Neal L. Weintraub, Ming Guo, Xin-Yun Lu

**Affiliations:** 1 Department of Neuroscience & Regenerative Medicine, Medical College of Georgia at Augusta University, Augusta, GA USA; 2 Binzhou Medical University Hospital, Shandong, China; 3 Department of Medicine, Vascular Biology Center, Medical College of Georgia at Augusta University, Augusta, GA USA

**Keywords:** Depression, Neuroscience

## Abstract

Social anhedonia, a loss of interest and pleasure in social interactions, is a common symptom of major depression as well as other psychiatric disorders. Depression can occur at any age, but typically emerges in adolescence or early adulthood, which represents a sensitive period for social interaction that is vulnerable to stress. In this study, we evaluated social interaction reward using a conditioned place preference (CPP) paradigm in adolescent male and female mice. Adolescent mice of both sexes exhibited a preference for the social interaction-associated context. Chronic unpredictable stress (CUS) impaired the development of CPP for social interaction, mimicking social anhedonia in depressed adolescents. Conversely, administration of leptin, an adipocyte-derived hormone, enhanced social interaction-induced CPP in non-stressed control mice and reversed social anhedonia in CUS mice. By dissecting the motivational processes of social CPP into social approach and isolation avoidance components, we demonstrated that leptin treatment increased isolation aversion without overt social reward effect. Further mechanistic exploration revealed that leptin stimulated oxytocin gene transcription in the paraventricular nucleus of the hypothalamus, while oxytocin receptor blockade abolished the leptin-induced enhancement of socially-induced CPP. These results establish that chronic unpredictable stress can be used to study social anhedonia in adolescent mice and provide evidence that leptin modulates social motivation possibly via increasing oxytocin synthesis and oxytocin receptor activation.

## Introduction

Anhedonia is one of the two core symptoms required for the diagnosis of major depression, the most common psychiatric disorder [1–3]. There are two main types of anhedonia, i.e., physical anhedonia and social anhedonia. While physical anhedonia represents a loss of interest or pleasure in physical experiences such as eating, touching, or sex, social anhedonia generally refers to an overall disinterest in social contact or a lack of pleasure in social engagement [4, 5]. Social anhedonia commonly manifests in adolescence, a critical period for social development that is highly vulnerable to stress [6, 7]. Suicide is the second leading cause of death for adolescents and is strongly associated with depression. Anhedonia predicts suicidal behavior and poor treatment response to antidepressant treatment [8, 9]. However, there is very limited research on anhedonia, especially social anhedonia, in adolescents. Lack of animal models that adequately recapitulate the core features of adolescent depression is the major hurdle.

The biological basis of depression remains elusive. The monoamine hypothesis has dominated our views on the pathophysiology of depression and the mechanisms of action of antidepressants for several decades [10]. Evidence also suggests a causal link between depression and a malfunction in the endocrine system. Indeed, endocrine hormones affect not only growth, development, metabolism, and stress responses, but also the course of mood disorders [11, 12]. Among these hormones, leptin, an adipocyte-derived hormone [13], plays an important role in key processes that take place during adolescence, including growth and pubertal development [14]. In humans, leptin concentrations increase throughout puberty in girls, whereas in boys leptin concentrations increase until the onset of puberty and then decline [15, 16]. Congenital leptin deficiency or leptin-receptor (LepR) deficiency leads to failure of pubertal development [17, 18], suggesting that leptin is essential for puberty to occur [19, 20]. In rodents, a dramatic increase in leptin concentrations occurs in the first two weeks of life, which is crucial for the establishment of the hypothalamic neural network and brain development [21, 22]. Mice deficient for leptin (ob/ob) or the leptin receptor (db/db) are infertile [17, 18]. Leptin administration can rescue the infertility phenotype of ob/ob mice [22, 23]. Our previous studies have shown that chronic stress reduces leptin levels in adult animals [24]. Systemic or intracerebroventricular (ICV) administration of leptin reverses chronic stress-induced depression-related behavioral deficits including anhedonia [24–29]. Moreover, human studies have found that lower serum and cerebroventricular leptin levels were associated with an increased risk of suicidal behavior [30–32]. However, whether leptin is a factor in determining adolescent vulnerability to chronic stress exposure and depression symptoms like social anhedonia is unknown.

Leptin exerts its effects by activating the long form of the LepRb, the major signaling component [33, 34]. LepRb is expressed in subpopulations of neurons in the brain [35, 36]. Within the hypothalamus, LepRb is abundant in several nuclei that control social behaviors, including the ventromedial hypothalamus, premammillary nucleus, and preoptic area [35–41]. Our previous studies have shown that acute leptin treatment promotes active social interaction and suppresses stress responses in adult male C57BL/6J mice [26]. In particular, leptin increases nosing and following behavior without affecting non-aggressive physical contact; it also suppresses self-grooming, a non-social behavior [26] that often occurs in response to a threat or a stressful environment [42–44].

In the present study, a conditioned place preference (CPP) paradigm induced by social interaction was used to determine social motivation in adolescent male and female mice. The impact of chronic unpredictable stress (CUS), a paradigm that induces depression-related behavioral deficits in adult mice [45–47], and leptin treatment on social motivation were examined. Furthermore, we explored the mechanisms that mediate leptin’s actions on social motivation, focusing on its functional interaction with the oxytocin system, which is well-known for its role in social behavior and social motivation [48–51].

## Methods and materials

### Animals

Male and female wild-type C57BL/6J adolescent mice (5–7-week-old) were either purchased from The Jackson Laboratory (Bar Harbor, MA, USA) or obtained from our in-house breeding colonies. LepRb:tdTomato reporter mice were produced as described previously [28]. Briefly, LepRb-IRES-Cre mice with an IRES-NLS-Cre cassette “knocked in” the region immediately 3′ to the LepRb stop codon were crossed with Ai14 (Gt(ROSA)26Sor^tm14(CAG-tdTomato^) mice that harbor a targeted mutation of the Gt(ROSA)26Sor locus with a loxP-flanked STOP cassette preventing transcription of a CAG promoter-driven tdTomato. The resulting offspring had the STOP cassette deleted by Cre-mediated recombination, enabling the expression of downstream tdTomato coding sequences in LepRb-expressing cells (LepRb-tdTomato). The offspring were genotyped using tail snipping with the following primers: Cre, forward-5′-CCAGCTAAACATGCTTCATCGTC-3′, reverse-5′- GGATTAACATTCTCCCACCGTCAG-3′; Ai14, forward (tdTomato)−5′-CTGTTCCTGTACGGCATGG-3′, reverse (WPRE)−5′-GGCATTAAAGCAGCGTATCC-3′. Mice were housed under a 12-h light-dark cycle (lights on at 06:00 AM) with ad libitum access to food and water. All animal procedures were conducted in accordance with NIH guidelines and approved by the Institutional Animal Care and Use Committee of Augusta University and Binzhou Medical University Hospital.

### Drugs

Recombinant mouse leptin (#498-OB, R&D Systems, Minneapolis, MN, USA) was dissolved in sterile saline at a concentration of 1.0 mg/ml and administered intraperitoneally (i.p.) at a dose of 1.0 mg/kg or 5.0 mg/kg body weight. For behavioral tests, leptin was administrated at a dose of 1.0 mg/kg, which has been used in our previous studies as an effective dose to produce behavioral effects including social interaction [25, 26, 28, 29, 52, 53]. A high dose of leptin (5.0 mg/kg) was chosen to determine its effects on gene expression, as it has been reported to effectively induce downstream signaling, i.e., p-STAT3 and p-AKT [54, 55]. A non-peptide selective oxytocin receptor antagonist (OTR-A), L-368,899 hydrochloride (#2641, Tocris Biosciences, Minneapolis, MN, USA) [56, 57], was dissolved in sterile saline at a concentration of 2.0 mg/ml and administered by i.p. injections at a dose of 5.0 mg/kg body weight [58].

### Socially conditioned place preference (CPP)

The protocol for socially conditioned place preference was adapted from previous studies [58–61]. C57BL/6 J male and female mice were weaned at 3 weeks of age and housed in a group of 3–5 per cage with corncob bedding (Teklad #7092) until behavioral testing. The CPP consisted of three phases including the pre-conditioning, conditioning, and test phases. In this study, CPP was a learned association between a condition (social contact or isolate) and a cue (bedding). At 6–7 weeks of age, mice were first subjected to a 30-min pre-conditioning trial to habituate to the apparatus and establish baseline preference to two types of novel bedding (sani-chips, Teklad #7090 and shredded aspen bedding, Teklad #7093) in a Plexiglas box (40 × 38 × 20 cm^3^) divided into two compartments of equal size by a clear Plexiglas wall with a 3.8-cm diameter circular hole at the bottom. Each chamber contained one type of novel bedding as preconditioned cues. Individual mice with strong baseline preference for either type of novel bedding (preference score >1.5) were excluded from further experimentation. The following day, mice were randomly assigned to a social cage with original cage mates to be conditioned to one type of novel bedding for 24 h, then singly housed in a cage to be conditioned to another type of bedding for 24 h. To assure an unbiased design, the assignments of bedding cues (novel bedding 1 vs novel bedding 2) were counterbalanced for two chambers (chamber A vs chamber B) as well as for two types of conditioning (social vs isolate). During the pre-conditioning trial, the assignments of bedding cues to two chambers (Chamber A and B) were as follows: novel bedding 1 in Chamber A and novel bedding 2 in Chamber B, or novel bedding 2 in Chamber A and novel bedding 1 in Chamber B. The first chamber and bedding exposure were randomly assigned to each mouse but kept consistent between pre- and post-conditioning trials. All mice in the same cage received the same treatment (i.e., control or CUS procedure, saline or leptin injection) and had the same bedding assignment (social vs isolate). Immediately after the isolation conditioning, mice were subjected to a 30-min post-conditioning test in the two-compartment box to determine preference for either type of bedding (social *vs* isolation). The time spent in each compartment was recorded during both the pre-conditioning session and post-conditioning test and analyzed by EthoVision XT (Leesburg, VA). Social CPP was evaluated by normalized social preference scores, which were derived by dividing the time spent in the social-paired compartment during the post-conditioning phase by the time spent in the same compartment during the pre-conditioning session. Subtracted social preference scores were derived by subtracting the time spent in the social-paired compartment during pre-conditioning session from the time spent in the compartment during post-conditioning test [58, 59].

To determine the contribution of social approach and isolation avoidance to the development of socially induced CPP [59, 61], the third type of novel bedding (laboratory pine shaving, Teklad #7088) was introduced during the conditioning phase. Following the pre-conditioning session in the two-compartment box with sani-chips and shredded aspen bedding in each compartment, mice were assigned to a social cage with cage mates to be conditioned to one type of bedding (i.e., sani-chips) for 24 h, and then singly housed in an isolation cage to be conditioned to another novel type of bedding (pine shaving) for 24 h. During the post-conditioning test, social approach was assessed by testing animals’ preference for social bedding (sani-chips) over neutral bedding (shredded aspen). Isolation avoidance was assessed by testing animals’ avoidance of isolation bedding versus neutral bedding.

### Chronic unpredictable stress (CUS)

As described previously [46, 47], male and female mice at 5 weeks of age were singly housed and received a variety of stressors at different times of the day (two stressors per day), including 2-h restraint, 15-min-tail pinch, 24-h disrupted light-dark cycle, 24-h wet bedding with 45° cage tilt, 10-min unpredictable and inescapable foot shock, and 30-min elevated platform. Control mice were briefly handled daily. The CPP procedure was performed at 24 h after exposure to the last stressor.

### RNA extraction and real-time PCR analysis

Adolescent male mice were sacrificed by rapid decapitation 15-min after i.p. injection of leptin (5.0 mg/kg). The brain was removed and sectioned coronally. Micropunch samples of the paraventricular nucleus (PVN) were obtained by using a 1.0-mm diameter puncher (Stoelting, Wood Dale, IL), snap frozen in liquid nitrogen after dissection, and stored at −80 °C until further processing for RNA extraction. Total RNA was extracted with the RNeasy Micro Kit (Qiagen #74004, Germantown, MD). On-column DNase I digestion was used to eliminate genomic DNA contamination. The quality and concentration of purified RNA were determined by NanoDrop™ 8000 Spectrophotometer (Thermo Fisher Scientific, Waltham, MA) and the High-Capacity cDNA Reverse Transcription Kit (Thermo Fisher Scientific #4368814, Waltham, MA) was employed to generate cDNA using random primers. The cDNA product was diluted and processed for real-time PCR quantification using the QuantStudio 5 real-time PCR system (Thermo Fisher Scientific, Waltham, MA) with the following primers: oxytocin hnRNA, forward-5′-CTGCGCTGCCAGGAGGAGAACTACCT-3′, reverse-5′-GGGTCAGTGTTCTGAGCTGCAAACCCG-3′; oxytocin mRNA, forward-5′-TGGCTTACTGGCTCTGACCT-3′, reverse-5′-GAGACACTTGCGCATATCCA-3′; β-tubulin, forward-5′-AGCAACATGAATGACCTGGTG-3′, reverse-5′-GCTTTCCCTAACCTGCTTGG-3′. As described previously [46, 62], the 2^(-ΔΔCT)^ method was used to obtain relative fold-change of gene expression normalized by β-tubulin compared with saline group.

### Immunohistochemistry

Immunohistochemistry was performed on brain sections from adolescent LepRb:tdTomato mice. Briefly, LepRb:tdTomato male mice were transcardially perfused under anesthesia through the ascending aorta using 0.1 M phosphate-buffered saline (PBS) followed by 4% paraformaldehyde (PFA) in PBS. The brains were removed and fixed overnight in 4% PFA and then transferred to 30% sucrose in PBS. Brains were cut into 40-μm coronal sections and stored in cryoprotectant (30% sucrose, 30% ethylene glycol, 1% polyvinyl pyrrolidone, 0.05 M sodium phosphate buffer) until processing for immunohistochemistry. Free-floating sections were first rinsed with 0.05 M Tris-buffered saline (TBS) and then incubated in 0.5% Triton-X 100 in TBS for 60 min, followed by incubation with a blocking buffer (10% donkey serum and 0.5% Triton-X 100 in TBS) for 1.5 h at room temperature. Brain sections were incubated with recombinant anti-oxytocin antibody in the blocking buffer (Abcam #ab212193, 1:2000, Cambridge, MA) overnight at 4 °C. The next day, brain sections were rinsed in TBS buffer four times (10 min each) and then incubated with donkey anti-rabbit IgG (H + L) secondary antibody conjugated to Alexa Fluor 488 (1:500, Thermo Fisher Scientific, Waltham, MA) at room temperature for 2 h. The sections were washed with TBS four times (10 min each) and then mounted on slides with an aqueous anti-fade mounting medium. KEYENCE All-in-One Fluorescence Microscope BZ-X800 (Keyence Corporation of America, Itasca, IL) was used to visualize the immunostaining and capture the images.

### Statistical analysis

All results are presented as mean ± s.e.m. (standard error of mean). Based on the mean and standard deviation of social CPP in our preliminary tests, a group size of *n* = 10 has 80% power to detect a 15% difference between groups with a standard deviation of 0.13 (α set at 0.05). Shapiro–Wilk test and F test were used to test the normality and equal variance assumptions, respectively. For normally distributed data, two-tailed *t* tests were used to assess differences between two experimental groups with equal variance and one-way analyses of variance (ANOVAs) followed by Sidak post hoc tests were used for analysis of three groups. For non-normally distributed data, Mann-Whitney nonparametric test was performed to compare two groups and the Kruskal-Wallis test followed by Dunn’s multiple comparisons test was used for comparison of multiple groups. The comparison of time spent in social or isolate bedding before and after conditioning was analyzed by two-tailed paired t-tests for normally distributed data or Wilcoxon matched-pairs signed rank test for non-normally distributed data. For all statistical analysis, *P*  <  0.05 was considered statistically significant.

## Results

### Chronic unpredictable stress impairs the formation of conditioned place preference for social interaction

**Fig. 1:**
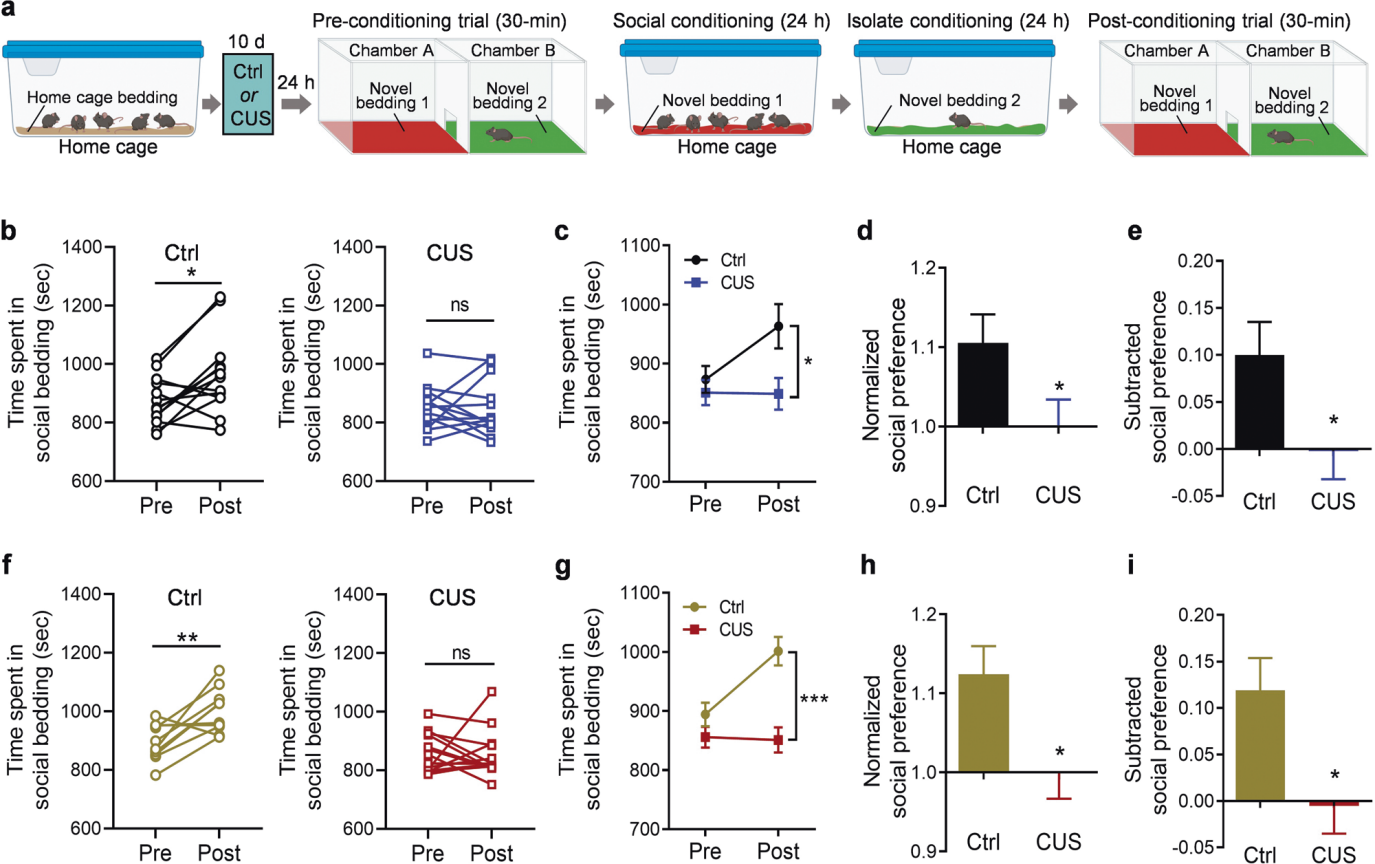
Chronic unpredictable stress impairs social interaction-conditioned place preference in adolescent mice. **a** Schematic diagram of the experimental design in control (Ctrl) mice and mice exposed to chronic unpredictable stress (CUS); **b**–**e** male mice (*n* = 13 mice per group), **f**–**i** female mice (Ctrl, *n* = 10; CUS, *n* = 14). Illustrations were created with BioRender.com. **b**, **f** Time spent in social bedding. **c**, **g** Average time spent in the compartment with social bedding. **d**, **h** Normalized social preference. **e**, **i** Subtracted social preference. *P <0.05, **P <0.01, ***P < 0.001 compared with pre-conditioning or control group; ns, no statistical significance.

Previous studies have shown that the magnitude of socially-induced CPP in C57BL/6 mice peaks at 6 weeks of age in males and 7 weeks of age in females [59]. Because sexual dimorphism has been reported in social behavior, especially in juvenile animals [63, 64], both male and female mice were included in the present study to test their preferences for cues (beddings) associated with social interaction at 6.5 weeks of age. Littermate mice were housed in groups for 2 weeks after weaning at 3 weeks of age, then underwent 10 days of the unpredictable stress procedure (CUS). After a preconditioning session to test baseline preference for two types of novel bedding, mice were conditioned first to associate one type of novel bedding with social housing for 24 h, then to associate the other type of bedding with isolation housing for 24 h, followed by a two-chamber, forced-choice preference assessment (Fig. 1a). Both male and female control mice displayed clear preferences for the bedding associated with social housing over the bedding associated with isolation housing (male mice: Fig. 1b, left, paired *t* test, *t*_(12)_ = 2.844, *P* = 0.015. female mice: Fig. 1f, left, paired *t* test, *t*_(9)_ = 3.419, *P* = 0.008). These results indicated that both male and female adolescent mice are able to form social CPP following a short-term, 2-day conditioning protocol. Thus, this paradigm can be used as a valid behavioral model to investigate the effects of chronic stress on social reward in adolescent mice.

**Fig. 2:**
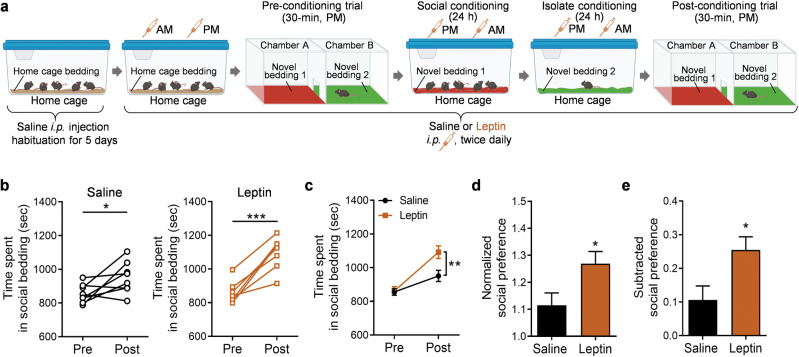
Leptin enhances social reward-conditioned place preference under non-stressed conditions in adolescent male mice. **a** Schematic diagram of the experimental design in saline control and leptin-injected (1.0 mg/kg) mice. Illustrations were created with BioRender.com. **b** Time spent in social bedding shown in individual mice. **c** Average time spent in the compartment with social bedding. **d** Normalized social preference. **e** Subtracted social preference. Saline treatment, *n* = 8 male mice; leptin treatment, *n* = 7 male mice. *P  <  0.05, **P  <  0.01, ***P  <  0.001 compared with pre-conditioning or saline-treated group.

To determine whether chronic stress can induce social anhedonia in adolescent mice, we used a chronic unpredictable stress paradigm, which can effectively induce depression-related behaviors in adult mice [46, 47]. Following two weeks of group housing after weaning, male and female mice were exposed to 10 days of unpredictable stress (two stressors per day) [46, 47]. To minimize the effects of acute exposure to stress, mice underwent the CPP procedure 24 h after the last stressor. Following two conditioning sessions to associate contextual cues (beddings) with social or isolation environment, mice of both sexes exposed to CUS showed no preference for the social-conditioned cue (Fig. 1b, right, Wilcoxon matched-pairs signed rank test, *P* = 0.722; Fig. 2f, right, Wilcoxon matched-pairs signed rank test, *P* = 0.426), reflecting a state of social anhedonia. Two-way ANOVA demonstrated a significant interaction between conditioning and chronic stress exposure (Male, Fig. 1c, stress × conditioning: *F*_(1, 24)_ = 4.918, *P* = 0.036, stress: *F*_(1, 24)_ = 4.194, *P* = 0.052, conditioning: *F*_(1, 24)_ = 4.484, *P* = 0.045; Female, Fig. 1g, two-way ANOVA analysis, stress × conditioning: *F*_(1, 22)_ = 7.354, *P* = 0.013, stress: *F*_(1, 22)_ = 19.51, *P* < 0.001, conditioning: *F*_(1, 22)_ = 6.132, *P* = 0.021). Moreover, the post-conditioning preferences for social bedding were expressed as normalized and subtracted social preference scores, both of which showed a decrease in social preference in mice exposed to CUS compared to control mice (Fig. 1d, *t*_(24)_ = 2.139, *P* = 0.043; Fig. 1e, *t*_(24)_ = 2.218, *P* = 0.036; Fig. 1h, Mann Whitney test, *P* = 0.013; Fig. 1i, Mann Whitney test, *P* = 0.013). These results suggest that social motivation in adolescent mice is sensitive to unpredictable stress.

### Leptin enhances social interaction-induced CPP in adolescent mice

Leptin has a permissive role in the initiation of puberty [17–20]. Our previous studies have shown that acute or sub-chronic treatment with leptin produces antidepressant-like effects in adult mice and rats [24–29]. To determine whether leptin modulates socially-induced CPP in adolescent mice, mice received i.p. injections (twice daily) of leptin (1.0 mg/kg) or vehicle (saline) during the pre-conditioning session and two conditioning sessions to associate two novel beddings with social interaction and isolation, respectively (Fig. 2a). To minimize the adverse effects of the injection procedure itself on conditioning and post-conditioning test, mice were injected with saline in the home cage for 5 days prior to the CPP procedure (Fig. 2a). While both saline-treated and leptin-treated groups showed place preference for the socially conditioned context (Fig. 2b; saline, paired t test, *t*_(7)_ = 2.535, *P* = 0.039; leptin, paired t test, *t*_(6)_ = 6.599, *P* = 0.0006), leptin-treated mice spent significantly more time in the chamber containing social bedding than saline-treated mice (Fig. 2c; treatment × conditioning: *F*_(1, 13)_ = 6.732, *P* = 0.022; treatment: *F*_(1, 13)_ = 5.076, *P* = 0.0422; conditioning: *F*_(1, 13)_ = 39.42, *P* < 0.001). Moreover, normalized and subtracted social preference data demonstrated a more robust place preference for social interaction in leptin-treated mice (Fig. 2d, *t*_(13)_ = 2.403, *P* = 0.032; Fig. 2e, *t*_(13)_ = 2.595, *P* = 0.022).

**Fig. 3:**
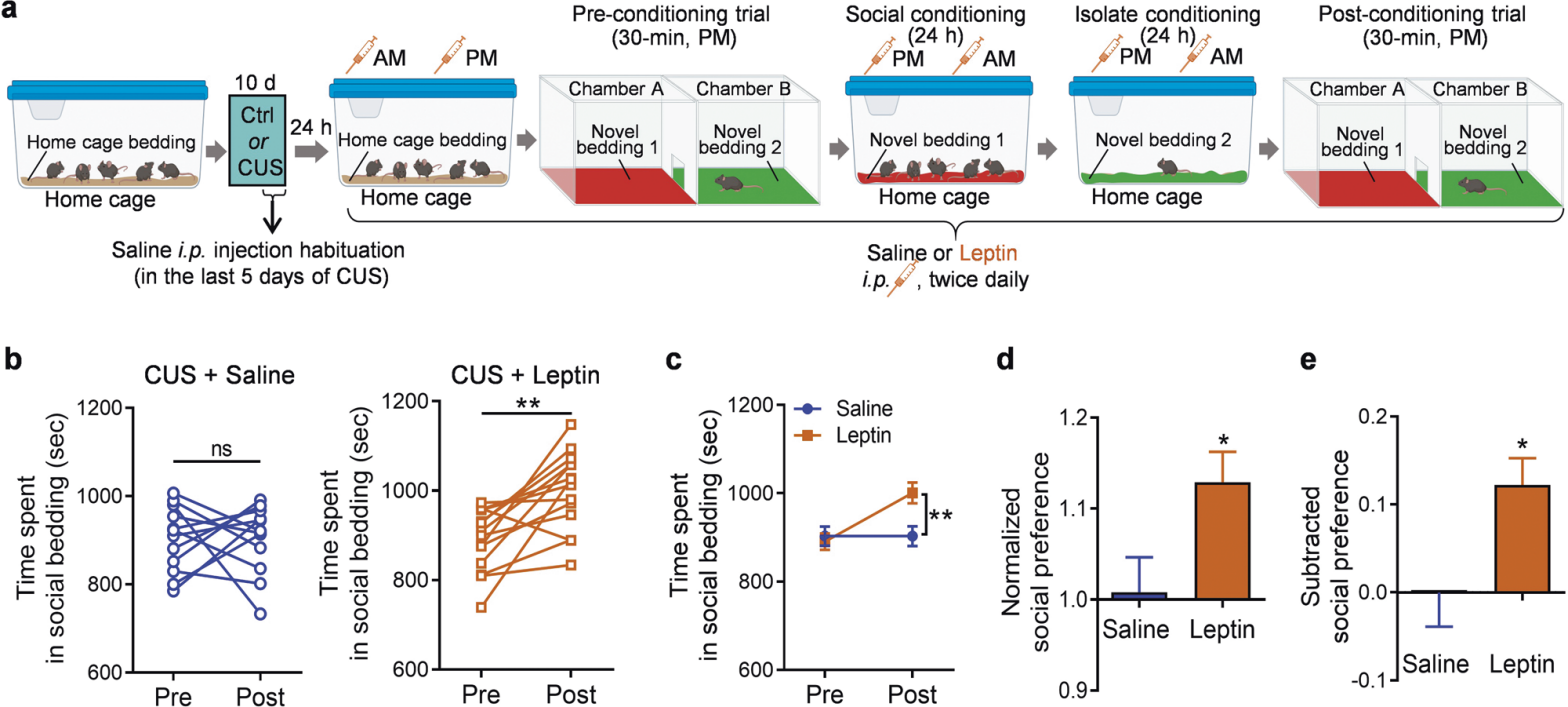
Leptin reverses chronic unpredictable stress (CUS)-induced social anhedonia in adolescent male mice. **a** Schematic diagram of the experimental design in saline control and leptin-injected (1.0 mg/kg) mice. Illustrations were created with BioRender.com. **b** Time spent in the compartment with social bedding. **c** Average time spent in the compartment with social bedding. **d** Normalized social preference. **e** Subtracted social preference. Saline treatment, *n* = 12 male mice; leptin treatment, *n* = 14 male mice. *P  <  0.05, **P  <  0.01 compared with pre-conditioning or saline-treated group; ns, no statistical significance.

Next, we asked whether the same leptin treatment regimen is sufficient to reverse CUS-induced social anhedonia. Male adolescent mice that were subjected to 10 days of CUS received i.p. injections of leptin in the home cage, social cage and isolation cage during pre-conditioning and conditioning sessions (i.p. 1.0 mg/kg, twice daily) (Fig. 3a). As observed in untreated mice exposed to CUS, mice exposed to CUS and treated with saline exhibited no preference for social bedding in the post-conditioning test (Fig. 3b, CUS + Saline, paired *t* test, *t*_(11)_ = 0.007, *P* = 0.994). In contrast, mice exposed to CUS and treated with leptin showed a significant preference for the socially conditioned context, as evidenced by more time spent in the chamber containing social bedding after conditioning (Fig. 3b, CUS + Leptin, paired *t* test, *t*_(13)_ = 4.023, *P* = 0.001). Two-way ANOVA demonstrated a significant interaction between conditioning and treatment (Fig. 3c; treatment × conditioning: *F*_(1, 24)_ = 6.353, *P* = 0.019; treatment: *F*_(1, 24)_ = 3.993, *P* = 0.0571; conditioning: *F*_(1, 24)_ = 6.296, *P* = 0.020). Leptin treatment significantly increased the normalized and subtracted social preference scores (Fig. 3d, *t*_(24)_ = 2.377, *P* = 0.026; Fig. 3e, *t*_(24)_=2.521, *P* = 0.019). These results indicate that leptin can reverse social anhedonia induced by chronic stress.

### Effects of leptin on social approach versus isolation avoidance in socially-induced CPP

**Fig. 4:**
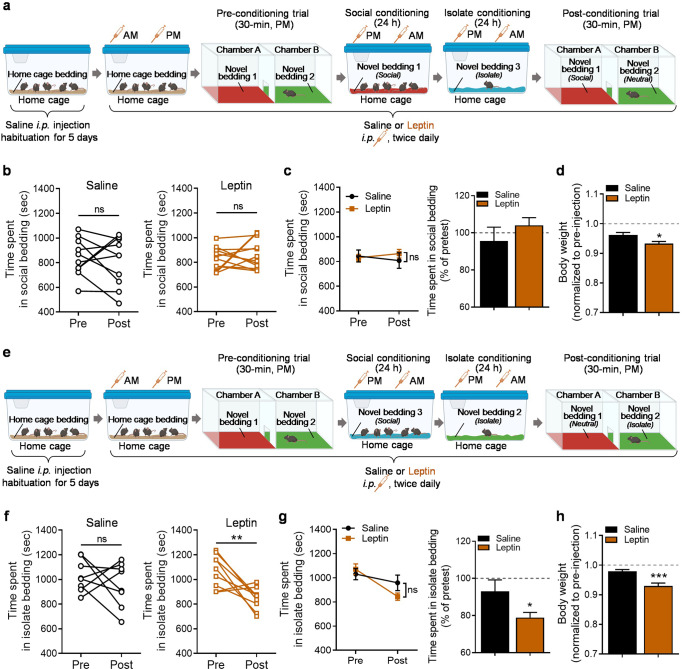
Effects of leptin treatment on social reward and isolation aversion in male mice. **a** Schematic diagram of the social reward experimental design in saline control and leptin-injected (1.0 mg/kg) mice. Illustrations were created with BioRender.com. **b** Time spent in the compartment with social bedding. **c** Left: average time spent in the compartment with social bedding; right: time spent in social bedding in post-trial normalized by pre-trial. **d** Body weight. Saline, *n* = 10 male mice; leptin, *n* = 12 male mice. **e** Schematic diagram of the isolation aversion experimental design in saline control and leptin-injected (1.0 mg/kg) mice. **f** Time spent in the compartment with isolate bedding. **g** Left: average time spent in the compartment with isolate bedding; right: time spent in isolate bedding in post-trial normalized by pre-trial. **h** Body weight. Saline, *n* = 8 male mice; leptin, *n* = 9 male mice. *P  <  0.05, **P  <  0.01 compared with pre-conditioning or saline-treated group; ns, no statistical significance.

The expression of socially induced CPP is attributed to two distinct motivational processes, i.e. social preference and isolation aversion [59, 61]. We thus investigated the effects of leptin on the social-conditioned cue versus the isolation-conditioned cue by introducing a third novel bedding; i.e., neutral bedding that was associated with neither social conditioning nor isolation conditioning. The preference assessed by a choice between social bedding and neutral bedding during the post-conditioning test would reflect social approach (Fig. 4a), whereas the preference assessed by a choice between isolation bedding and neutral bedding would be driven by isolation withdrawal (Fig. 4e). In contrast to the reported 10-day conditioning procedure [61], the present study used a short-term, 2-day conditioning procedure [58, 59]. Using this paradigm, neither component, *i.e*. social approach or isolation withdrawal, was sufficient to induce place preference independently in mice treated with saline (Fig. 4b, saline injection, paired *t* test, *t*_(9)_ = 0.588, *P* = 0.571; Fig. 4f, saline injection, paired *t* test, *t*_(7)_ = 0.811, *P* = 0.444). However, while leptin treatment (i.p. 1.0 mg/kg, twice daily) showed no effect on the social approach-driven preference (Fig. 4b, leptin injection, paired *t* test, *t*_(11)_ = 0.754, *P* = 0.467. Figure 4c left, treatment × conditioning: *F*_(1, 20)_ = 0.878, *P* = 0.360; treatment: *F*_(1, 20)_ = 0.1948, *P* = 0.664; conditioning: *F*_(1, 20)_ = 0.002, *P* = 0.962. Figure 4c right, *t*_(20)_ = 1.030, *P* = 0.3152), the social isolation-driven withdrawal was increased by leptin, as indicated by a decrease in time spent in isolation-conditioned context (Fig. 4f, leptin injection, paired *t* test, *t*_(8)_ = 3.516, *P* = 0.008; Fig. 4g left, treatment × conditioning: *F*_(1, 15)_ = 2.030, *P* = 0.175, treatment: *F*_(1, 15)_ = 0.964, *P* = 0.342, conditioning: *F*_(1, 15)_ = 7.624, *P* = 0.015). The time spent in the isolate bedding during the post-conditioning trial was normalized to the pre-conditioning trial. After normalization, time spent in the chamber with the isolate bedding was significantly decreased by leptin treatment compared with saline treatment (Fig. 4g right, *t*_(15)_ = 2.161, *P* = 0.047). These results demonstrate that leptin promotes avoidance of environmental cues associated with social isolation. Compared with the saline-treated group, leptin treatment significantly decreased body weight, confirming efficacy of leptin (Fig. 4d, *t*_(20)_ = 2.764, *P* = 0.012; Fig. 4h, *t*_(15)_ = 4.141, *P* = 0.0009).

### Leptin increases oxytocin transcription in the paraventricular nucleus of hypothalamus

**Fig. 5:**
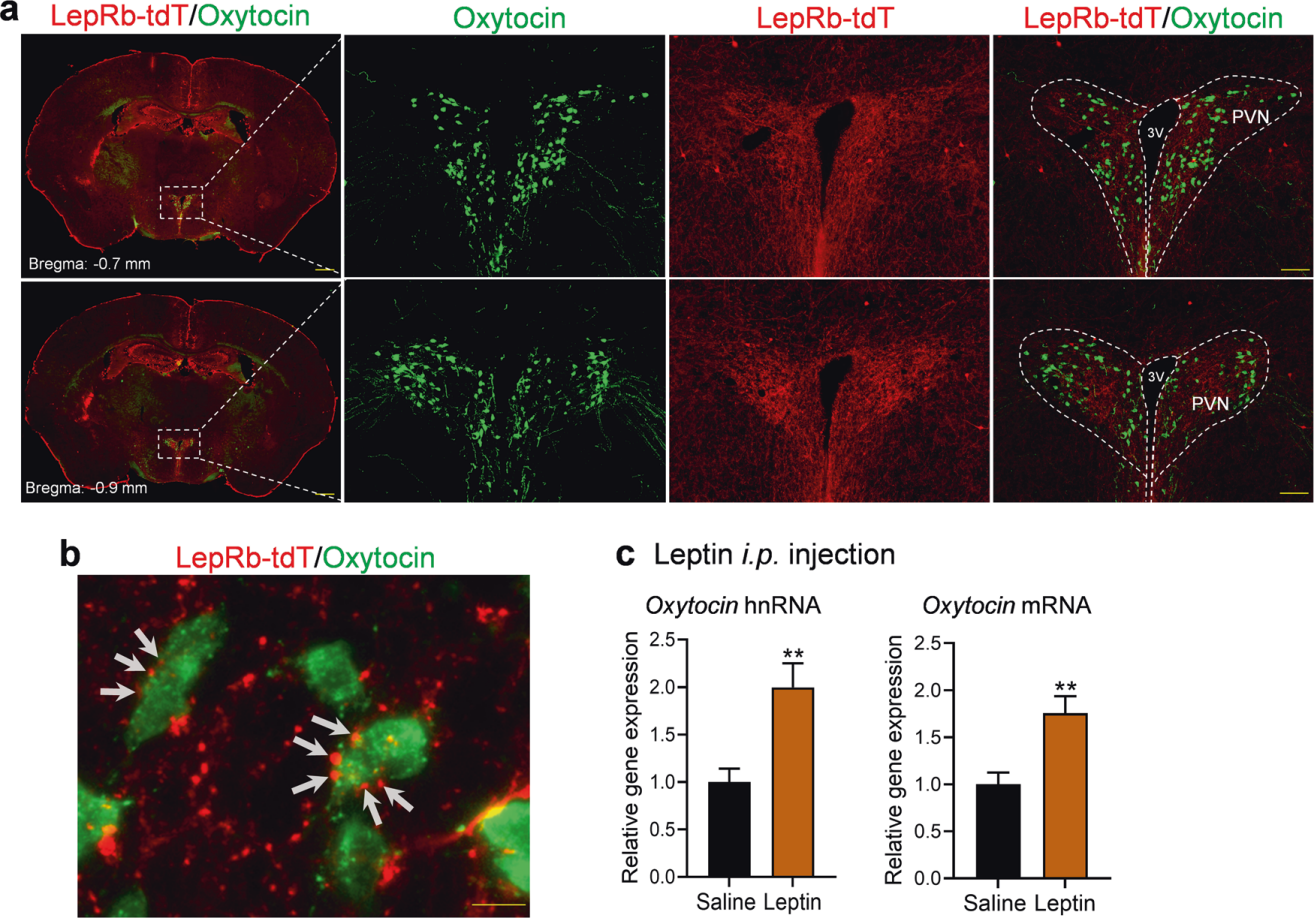
Leptin induces oxytocin expression in the paraventricular nucleus (PVN) of the hypothalamus in male mice. **a** Distribution of leptin receptor (LepRb)-expressing cells (red) and oxytocin-immunoreactive neurons (green) in the different levels of PVN. Scale bars, 500 µm for low magnification and 100 µm for high magnification. **b** High-magnification image showing close apposition of puncta of LepRb-expressing neurons with oxytocin-positive neurons. Scale bar, 10 µm. **c** Oxytocin RNA expression levels 15 min after i.p. leptin injection. Left, hnRNA expression levels; right, mRNA expression levels. *n* = 8 male mice per group. **P  <  0.01 compared with saline-treated group.

Next, we explored potential neural substrates that mediate leptin’s effects on social CPP. Oxytocin plays a key role in mediating social interaction [48–51]. To test the hypothesis that oxytocin mediates leptin’s effects on social CPP, we examined both anatomical and functional interactions between leptin signaling and the oxytocin system. To provide an anatomical basis for oxytocin neurons as a target of leptin, we attempted to probe for co-localization of LepRb in oxytocin neurons using a LepRb:tdTomato reporter line. While only a few sparsely distributed LepRb-expressing neurons were present in the PVN and showed no colocalization with oxytocin neurons (Fig. 5a), the PVN contained a dense terminal cluster of LepRb-expressing neurons, suggesting that oxytocin neurons could be an indirect target of leptin action (Fig. 5a). Indeed, we found that LepRb-positive terminals were present in close proximity to oxytocin neurons in the PVN (Fig. 5b). To test whether leptin may regulate oxytocin expression, mice received an i.p. leptin injection (5.0 mg/kg) 15 min prior to collecting the PVN tissue punches to detect heterogeneous nuclear (hn)RNA and mRNA levels. Both hnRNA and mRNA levels of oxytocin were increased in the PVN by leptin (hnRNA: *t*_(14)_ = 3.426, *P* = 0.004; mRNA: *t*_(14)_ = 3.410, *P* = 0.004), suggesting that leptin can acutely stimulate oxytocin gene transcription.

### Blockade of oxytocin receptors abolishes the leptin enhancement of socially-induced CPP

**Fig. 6:**
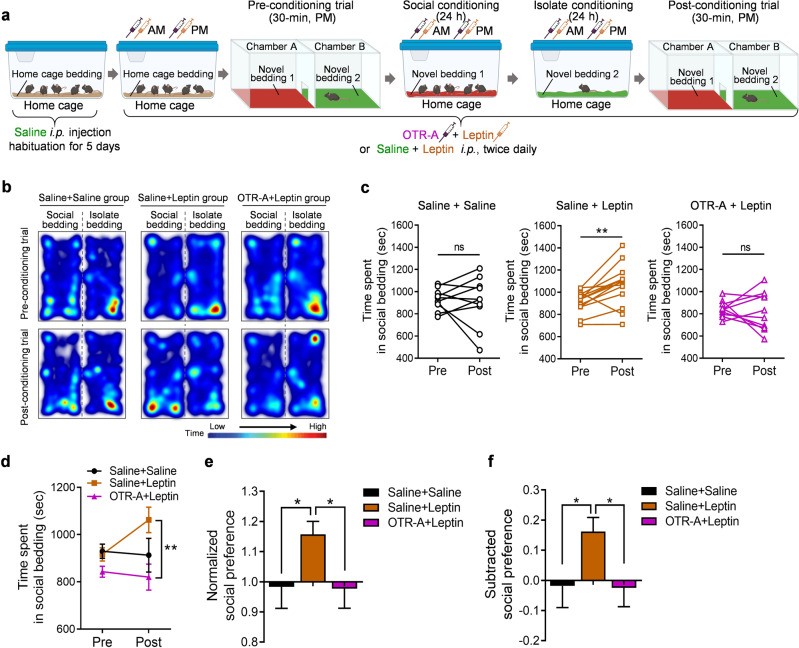
Blockade of oxytocin receptors abolishes leptin-induced enhancement of social preference. **a** Schematic diagram of the experimental design. Illustrations were created with BioRender.com. **b** Representative images of heat maps depict the time spent by the mice from three treatment groups in the chamber containing social bedding versus the chamber containing isolate bedding during the pre-conditioning trial (upper panel) and post-conditioning trial (lower panel). **c** Time spent in the compartment with social bedding. **d** Average time spent in the compartment with social bedding. **e** Normalized social preference. **f** Subtracted social preference. Saline + Saline, n = 10 male mice; Saline + Leptin, *n* = 13 male mice; OTR-A + Leptin, *n* = 10 male mice. *P  <  0.05, **P  <  0.01 compared with relative control groups; ns, no statistical significance. OTR-A, oxytocin receptor antagonist L-368,899.

To determine whether oxytocin signaling mediates the effects of leptin on socially-induced CPP, mice were pretreated with an OTR-A, L-368,899 hydrochloride (5.0 mg/kg, i.p.) 30 min before each leptin injection during pre-conditioning and conditioning sessions for 3 consecutive days (Fig. 6a). L-368, 899 hydrochloride can penetrate the blood–brain barrier and has been reported to accumulate in the limbic brain areas after systemic administration [65]. While L-368,899 hydrochloride is a potent, non-peptide oxytocin receptor antagonist (IC_50_ = 8.9 nM), it can also bind to vasopressin V_1a_ and V_2_ receptors but with much lower affinity (IC_50_ = 370 nM for V_1a_ and 570 nM for V_2_, respectively). In this study, we used a relatively low dose of L-368,899 hydrochloride, 5.0 mg/kg, for i.p. injection. At this concentration, L-368,899 hydrochloride would preferentially bind to the high-affinity oxytocin receptor in the brain. Saline-treated mice failed to form place preference for social bedding (Fig. 6b, c, Saline + Saline injection, paired t test, *t*_(9)_ = 0.258, *P* = 0.802), which might be due to the stress effects of repeated injections (4 i.p. injections per day for 3 days). Leptin-treated mice (2 saline i.p. injections/2 leptin i.p. injections per day for 3 days) exhibited significant social CPP (Fig. 6b, c, Saline + Leptin injection, paired *t* test, *t*_(12)_ = 3.481, *P* = 0.0045). This effect of leptin was blocked by pretreatment with OTR-A (2 OTR-A i.p. injections/2 leptin i.p. injections) (Fig. 6b, c, OTR-A + Leptin injection, paired *t* test, *t*_(9)_ = 0.398, *P* = 0.700). Two-way ANOVA analysis demonstrated a significant interaction between treatment and conditioning (Fig. 6d; treatment × conditioning: *F*_(2, 30)_ = 3.467, *P* = 0.044; treatment: *F*_(2, 30)_ = 4.350, *P* = 0.022; conditioning: *F*_(1, 30)_ = 1.325, *P* = 0.2588). The normalized and subtracted social preference scores confirmed a significant enhancement of social CPP induced by leptin treatment, and the elimination of this effect by blocking oxytocin receptors (Fig. 6e, one-way ANOVA, *F*_(2, 30)_ = 3.299, *P* = 0.0507; Fig. 6f, one-way ANOVA, *F*_(2, 30)_ = 3.468, *P* = 0.044). These results suggest that leptin modulates social CPP by activating the oxytocin system.

## Discussion

Social interactions are rewarding for many different species, including humans and mice [58, 61, 66]. The present study used a conditioned place preference paradigm to measure the rewarding effects of social interaction and social reward learning in adolescent mice. We found both male and female adolescent mice display a conditioned social preference. Chronic exposure to unpredictable stress impairs acquisition of social CPP after conditioning, reflecting a state of social anhedonia. Conversely, leptin treatment enhances social CPP and reverses CUS-induced social anhedonia. Furthermore, we demonstrate that leptin-enhanced social CPP is mediated by activating the oxytocin signaling pathway.

Adolescence is the period of transition between childhood and adulthood, from ages 10 to 19 in humans. It is a unique stage of human development, involving dramatic physical, hormonal, behavioral, and neurobiological changes. However, the exact age span that encompasses adolescence in mice is not precisely defined. It is generally considered to start with the onset of puberty, a process leading to sexual maturation, which is strain- and sex-dependent and influenced by environmental factors [67–71]. Similar to humans, pubertal onset in female mice can occur earlier than that of males [70, 71]. The onset of puberty is characterized by specific physiological and hormonal changes [70, 72]. In C57BL/6 mice, pubertal onset starts at P28 in female mice and P30 in male mice [70, 73, 74]. Female mice typically become sexually mature at P42 (6 weeks) and males at P56 (8 weeks) [75]. Adolescence is a critical period for social development. Social play behavior is a highly rewarding activity in young animals [76–78], which can be used to induce conditioned place preference (i.e., CPP for social interaction) [61, 79]. In mice, social play behavior begins around the time of weaning and maintains similar patterns across adolescent development in both male and female mice [61, 70]. Although female mice reach sexual maturity at an earlier age, they form socially induced CPP at a later age compared to male mice. Social interaction-induced conditioned CPP in C57/BL/6 mice peaks at 6 weeks of age in males and 7 weeks of age in females [59]. Here, we used both male and female mice to determine the effects of exposure to 10 days of unpredictable stress (i.e. CUS) on social CPP, which was conducted at 6.5 weeks of age. We demonstrated for the first time that chronic stress impairs preference for social engagement after conditioning in adolescent mice, mimicking social anhedonia [4, 5]. However, it is possible that the potential influence of stress exposure on social interaction during the conditioning session may affect the formation of a conditioned preference for social interaction.

Depression can occur at any age, but usually emerges in adolescence or early adulthood, which represents a sensitive period for social interaction that is more vulnerable to stress [6, 7]. Our findings suggest that CUS can be used to induce social anhedonia in adolescence. Given that anhedonia is a core symptom of depression [3], an anhedonic phenotype should be included as a key feature of animal models of depression. Although chronic mild/variable/unpredictable stress has been widely used to induce anhedonia, the majority of previous studies have focused on hedonic deficits in response to food- and sex-related stimuli as measured by sucrose preference and female urine sniffing tests [80–82].

We have previously shown that acute leptin treatment promotes active social interaction and suppresses stress responses in adult male C57BL/6J mice [26]. In light of our previous observations, we administered leptin to adolescent mice during pre-conditioning, social conditioning, and isolation conditioning to test its effects on conditioned social preference. We found that leptin treatment enhanced place preference for the socially conditioned context in non-stressed adolescent male mice. In adolescent mice subjected to 10 days of CUS, leptin treatment was able to restore preference for social engagement. This finding is consistent with our previous reports that leptin treatment can reverse CUS-induced anhedonia in the sucrose preference test in adult animals [24, 27]. Social preference in the present study was assessed using a forced choice between two chambers, with the environmental cues associated with social interaction and social isolation, respectively. Thus, the preference for the socially-conditioned context could be driven by either social approach or isolation avoidance [59, 61]. By introducing a neutral cue, mice were offered a choice between the socially associated cue and the neutral cue to assess social approach and a choice between the isolation-associated cue and the neutral cue to assess isolation avoidance. Due to short-term conditioning (1-day conditioning for social interaction followed by 1-day conditioning for isolation), mice failed to show overt preference for social cues or significant aversion for isolation cues. This is in line with a previous report that mice without social deprivation display only moderate social CPP in early adolescence [83, 84]. Previous studies have reported that a longer term of conditioning (10 days) is required for mice to develop conditioned place preference [61]. However, while leptin treatment had no effect on preference for social cues, aversion to isolation cues was increased by leptin in adolescent male mice, suggesting that social approach and isolation avoidance have differential sensitivity to leptin treatment. We and others have shown that estrogens influence central leptin sensitivity [25, 85, 86]. We found that adult female mice displayed estrous cycle-dependent behavioral responses to leptin [25]. Determination of estrous cycle phases using vaginal smears is a stressful procedure that could alter behavioral responses and estrous cycles. Our previous studies used adult female mice that displayed at least two consecutive 4-day estrous cycles prior to behavioral testing. However, the length of time required for the confirmation of normal estrous cycles is not compatible with the time window for conditioned social preference in adolescent mice to investigate leptin’s effects in female mice.

Oxytocin plays a key role in modulating social behaviors [48–51]. While oxytocin neurons are located in both the PVN and the supraoptic nucleus (SON) in the hypothalamus [87, 88], oxytocin neurons in the PVN, but not SON, are crucial for social reward [58, 89, 90]. Oxytocin neurons in the PVN are activated during social interaction [58, 91–93]. Stimulation of oxytocin neurons in the PVN (or hypothalamus) is sufficient to induce several types of social behaviors, such as social preference, social recognition, and social bonding [92, 94, 95], whereas systemic injections of oxytocin receptor antagonists can block social CPP [58]. Evidence suggests that oxytocin neurons in the PVN may be the major target for leptin to regulate social behavior. ICV leptin injection has been shown to activate PVN oxytocin neurons in fasted rats, as indicated by c-Fos induction in identified oxytocin neurons [96–98]. Also, ICV leptin injection was found to induce STAT3 phosphorylation in PVN oxytocin neurons in fasted rats, and repeated leptin injections during fasting increased oxytocin expression [96]. However, we found that i.p. injection of leptin in mice induced c-Fos only in a few oxytocin neurons scattered throughout the PVN (data not shown). This is consistent with our observations of sparsely distributed LepRb-expressing neurons in the PVN and the absence of co-localization of LepRb and oxytocin. Nevertheless, the close apposition of LepRb-positive puncta on the soma and processes of oxytocin-positive neurons indicates the presence of presynaptic fibers from LepRb-expressing neurons impinging on oxytocin neurons, suggesting that they are situated in synapses where LepRb activation may influence oxytocin neuron functions. We found that systemic leptin treatment induces a rapid increase in oxytocin hnRNA and mRNA levels in the PVN. The steady-state mRNA levels are determined by transcription and degradation processes. Because of the rapid turnover of the primary transcript and intermediate forms of RNA in the nucleus, a measurement of hnRNA levels primarily reflects the transcription rate of the gene and the level of encoded peptide synthesis [99]. Given that pretreatment with the oxytocin receptor antagonist L-368,899 hydrochloride prior to each leptin administration blocked leptin-induced enhancement of social CPP, activation of oxytocin signaling may be involved in the mechanisms underlying leptin action on social reward and social learning.

Evidence suggests that oxytocin gates information flow into reward circuits via specific pathways. Oxytocin neurons in the PVN directly project to the nucleus accumbens and the ventral tegmental area (VTA), where oxytocin is released from presynaptic terminals and activates oxytocin receptors to generate social reward [58, 89, 100–103]. Direct infusion of oxytocin into the VTA enhanced the activity of dopamine neurons, potentiated social reward, and modulated reward-related behavior [104, 105]. Conversely, inhibition of VTA-projecting PVN oxytocin neurons, or deletion of oxytocin receptors in VTA dopamine neurons, impaired the formation of social CPP [89]. In the nucleus accumbens, local infusion of an oxytocin receptor antagonist to block presynaptic receptors arising from dorsal raphe 5-HT neurons can prevent social CPP [58]. These studies suggest that oxytocin neurons in the PVN, and oxytocin action at two projection sites, i.e. the VTA and the nucleus accumbens, are required for social award. Leptin may regulate oxytocin synthesis in PVN neurons and/or modulate the activity of PNV oxytocin neurons projecting to the VTA and the nucleus accumbens. Oxytocin neurons in the PVN receive projections from hypothalamic nuclei containing leptin target neurons [35, 36], including glutamatergic and GABAergic innervation from the ventromedial hypothalamus [106, 107] and preoptic area [107, 108], which has been shown to control social behaviors [37–41]. Thus, leptin may presynaptically modulate synaptic transmission between its target neurons and oxytocin neurons, contributing to the regulation of social behavior. Another possibility is that leptin could influence oxytocin action postsynaptically in these two projection sites. While lack of co-localization of LepRb in 5-HT neurons does not support a leptin-oxytocin interaction at 5-HT presynaptic terminals in the nucleus accumbens [109], VTA dopamine neurons express LepRb and oxytocin receptors, and their firing activity is modulated by both leptin and oxytocin [53, 54]. Whether leptin and oxytocin converge on VTA dopamine neurons, and leptin regulates social behavior via modulating oxytocin receptor activity in the VTA, requires further investigation.
